# A retrospective study of hip posterior fracture-dislocation: closed reduction at the emergency department or in the operation theater?

**DOI:** 10.1186/s10195-022-00677-0

**Published:** 2022-12-02

**Authors:** Po-Ju Lai, Chih-Yang Lai, I-Chuan Tseng, Chun-Yi Su, Yung-Heng Hsu, Ying-Chao Chou, Yi-Hsun Yu

**Affiliations:** 1 Division of Orthopedic Traumatology, Department of Orthopedic Surgery, Musculoskeletal Research Center, Chang Gung Memorial Hospital, 33302 Tao-Yuan City, Taiwan; 2 The Department of Orthopaedic Surgery, Chang Gung Memorial Hospital, Taoyuan branch, Tao-Yuan City, Taiwan; 3 The Department of Orthopaedic Surgery, Chang Gung Memorial Hospital, Keelung branch, Keelung City, Taiwan

**Keywords:** Closed reduction, Hip posterior fracture-dislocation, Emergency department, Operating theater

## Abstract

**Background:**

For hip posterior fracture-dislocation, the current consensus is to perform joint reduction within 6 h to prevent sequelae. However, whether a closed reduction (CR) should be performed at the emergency department (ED) or in the operation theater (OT) remains debatable. We aimed to assess the incidence and factors predictive of CR failure at the ED in patients with hip posterior fracture-dislocation.

**Methods:**

Patients with hip posterior fracture-dislocation between 2009 and 2019 were included. Age, sex, body mass index (BMI), injury severity score, new injury severity score, time from injury to first reduction attempt (TIR), presence of associated femoral head fracture, posterior wall marginal impaction, and posterior wall fragment size were compared between patients with CR success and patients with CR failure at the ED.

**Results:**

Fifty-five patients with hip posterior fracture-dislocation experienced CR attempts at the ED and were enrolled in the study. Thirty-eight (69.1%) hips were reduced successfully at the ED, and 17 (30.9%) experienced failure. No significant differences in age, sex, BMI, presence of femoral head fracture, marginal impaction, or size of the posterior wall fragment were found between the groups. TIR was significantly shorter in the successful CR group (2.24 vs. 4.11 h, *p* = 0.01). According to receiver operating characteristic curve analysis, 3.5 h was the cut-off time.

**Conclusions:**

For patients with hip posterior fracture-dislocation, TIR was a critical factor for successful CR. If the time interval exceeds 3.5 h from injury, the success rate of bedside CR at the ER is likely to decrease, and the OT should be prepared in case of failed bedside CR.

*Level of Evidence* III.

## Introduction

For hip posterior fracture-dislocation, the current consensus is to perform joint reduction as soon as possible. A delayed reduction of a dislocated hip may lead to an increased incidence of early sequelae such as avascular necrosis of the femoral head or post-traumatic osteoarthritis [1–8]. Recent evidence suggests that the optimal reduction time for a dislocated hip is within 6 h from dislocation [9–11], whether by the closed or open method.

Closed reduction (CR) is often the first-line treatment as it can be performed in the emergency department (ED) under procedural sedation and analgesia or in the operation theater (OT) under general anesthesia. The advantages of CR in the ED include it being a cost-effective and a time-saving procedure [12–14]. However, some factors, such as obesity, the presence of a femoral head fracture [15], having a large muscle mass, and having a femoral head perched on the acetabular rim [16], may increase the difficulty of bedside CR and jeopardize the normal tissue during forceful CR. These patients may benefit from dislocated hip joint reduction under general anesthesia with endotracheal tube intubation and proper muscle relaxation, which is safer in an OT setting. Additionally, a CR can be changed to open reduction (OR) when facing an irreducible hip joint by CR under general anesthesia.

Multiple CR attempts may delay timely treatment, increase patient discomfort, and cause iatrogenic fracture of the proximal femur; thus, we investigated whether an irreducible hip joint can be predicted. Our study aimed to analyze factors that might potentially have negative effects on the success rate of CR in the ED. By identifying these factors, we can perform the reduction maneuver in the OT rather than in the ED to save time and reduce procedure-related complications.

## Materials and methods

### Patients

We retrospectively reviewed patients with hip posterior fracture-dislocation from the trauma registration of a level 1 trauma center from 2009 to 2019 to identify factors that might affect the success rate of CR in the ED. We included adult patients presenting with hip posterior fracture-dislocation who underwent the first CR attempt in the ED. Patients less than 18 years old or with a dislocated hip that had already been reduced before arrival at our ED were excluded from the study. The review process was approved by our institutional review board (no: 202101823B0), and the requirement for informed consent was waived owing to the retrospective nature of this study. The study was performed in accordance with the Declaration of Helsinki.

### Resuscitation and treatment protocol

All patients followed the treatment protocol for hip fracture dislocation in our hospital. Initial resuscitation and primary survey were initiated upon arrival at our ED. For those who were unconscious and in a state of shock, complained of hip pain, and presented abnormal hip rotation and shortening of the lower extremities, a standard pelvic radiographic evaluation in the anteroposterior (AP) view was done. Once the hip joint dislocation was confirmed, the reduction was promptly performed.

For patients who had life-threatening conditions (head, chest, or abdominal injury) or other orthopedic emergencies (Gustilo type III open fracture, compartment syndrome, or active bleeding) that needed an immediate operation, the patient was sent to the OT directly for simultaneous operation and CR of the hip joint under general anesthesia.

However, if the patient had a stable hemodynamic status, CR was performed by an orthopedic surgeon at the bedside in the ED. Procedure sedation and analgesia with two medications, thiamylal sodium 300 mg (Citosol, Shinlin Sinseng Pharmaceutical Co. Ltd., Taoyuan City, Taiwan) and morphine HCl 10 mg (Bureau of Controlled Drugs, Taiwan Food and Drug Administration), were achieved intravenously. With adequate sedation and analgesia, reduction using different techniques was attempted. The reduction maneuver used was based on the preference of the in-charge orthopedic surgeon (senior orthopedic resident), mostly with a combination of the Allis, Lefkowitz, and Captain Morgan maneuvers [17]. Once the reduction was achieved, post-reduction pelvic radiography in the AP view of the pelvis was performed for confirmation. A three-dimensional reconstructed computed tomography scan of the hip was subsequently performed to better evaluate the presence of intra-articular osteochondral fragments, marginal impaction, and associated femoral head fracture for subsequent surgical planning.

If CR could not be achieved at the bedside within the therapeutic time of the sedation and analgesia, the patient was sent to the OT for reduction under general anesthesia, with endotracheal intubation and proper muscle relaxation. Fluoroscopy was also used sometimes to assist CR in the OT. If CR failed, OR was performed to reduce the dislocated hip. The Kocher–Lagenbeck approach was preferred to reduce the dislocated hip, and osteosynthesis was performed simultaneously. Occasionally, a greater trochanteric osteotomy was used to address the femoral head lesion.

### Data collection and statistical analysis

We collected data including age, sex, body mass index (BMI), injury severity score (ISS), new ISS (NISS), time from injury to first reduction attempt (TIR), presence of associated femoral head fracture, posterior wall marginal impaction, and posterior wall fragment size (calculated with Moed’s method) [18]. Data were analyzed using SPSS software (version 26.0; SPSS Inc., Chicago, IL, USA). Continuous variables were compared using Student’s *t*-test, and categorical variables were compared using the chi-squared test and Fisher’s exact test. Statistical significance was set at a *p* value of < 0.05.

## Results

**Fig. 1:**
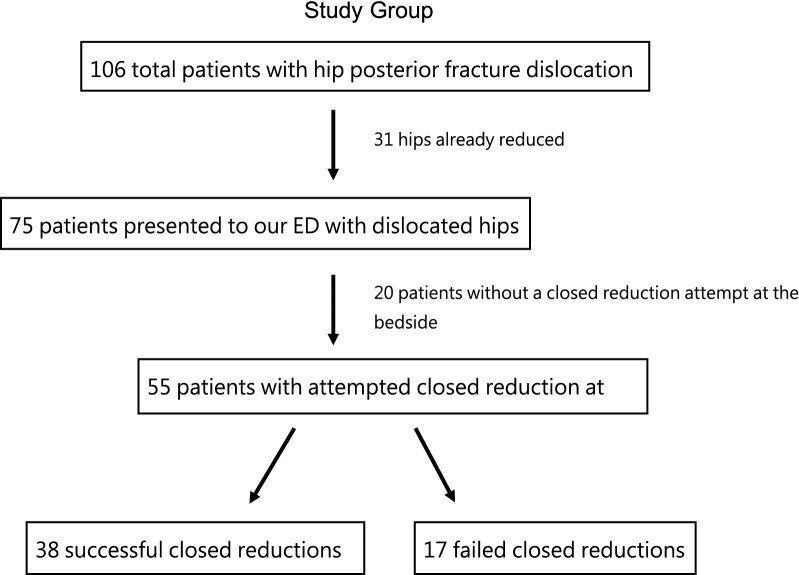
Study group inclusion/exclusion tree. *ED* emergency department

From 2009 to 2019, 106 patients who experienced hip fracture-dislocation were sent to our hospital. Of these patients, 75 arrived at our ED with their hips still dislocated. After resuscitation and the primary survey, 20 patients were sent to the OT for emergency operation without attempting CR at the bedside at the ED. The emergency operations were performed for intracranial hemorrhage in 6 patients, blunt abdominal trauma with internal bleeding for 4 patients, and type III open fracture that needed debridement or hemostasis for 14 patients. These 20 patients successfully received CR for the dislocated hip under general anesthesia at the OT (Fig. 1). Additionally, 8 patients were intubated because of cranial and torso injuries during hospital transfer or at the ER, but they were excluded from the analysis. After the exclusion of these 20 patients, 55 patients were enrolled in the study for analysis.

The demographic data of the enrolled patients are shown in Table 1. Among the 55 patients, 38 (69.1%) underwent successful hip reduction at the bedside and subsequently underwent osteosynthesis. In 17 (30.9%) patients, the reduction attempts at the bedside failed, and these patients were sent to the OT for CR under general anesthesia with endotracheal tube intubation. Three (5.5%) patients eventually needed OR through the Kocher–Lagenbeck approach with simultaneous osteosynthesis. No procedure-related complications were reported during CR, either in the ED or OT.

**Table 1:** Characteristics of patients receiving closed reduction attempts for hip posterior fracture-dislocation at our ED (2009–2019)

Case number	55
Age (mean + SD) years	34.52 (SD 14.81)
Sex (%)	
Male	46 (83.6%)
Female	9 (16.4%)
ISS (mean + SD)	11.91 (SD 6.09)
NISS (mean + SD)	19.18 (SD 6.95)
BMI (mean + SD)	27.23 (SD 5.67)
Injury mechanism (%)	
MVA, motorcycle	44 (80.0%)
MVA, car	10 (18.2%)
Fall from height	1 (1.8%)
Marginal impaction (%)	18 (32.7%)
Associated femoral head fracture (%)	23 (41.8%)
Posterior wall fragment size (%, mean + SD)	33.43 (SD 19.60)
Time to first closed reduction attempt (h, mean + SD)	2.82 (SD 1.70)
Time to reduction (h, mean + SD)	3.81 (SD 3.34)

*ISS* injury severity score, *NISS* new injury severity score, *BMI* body mass index, *MVA* motor vehicle accident, *ED* emergency department, *SD* standard deviation. Posterior wall fragment size was based on Moed’s method

**Fig. 2:**
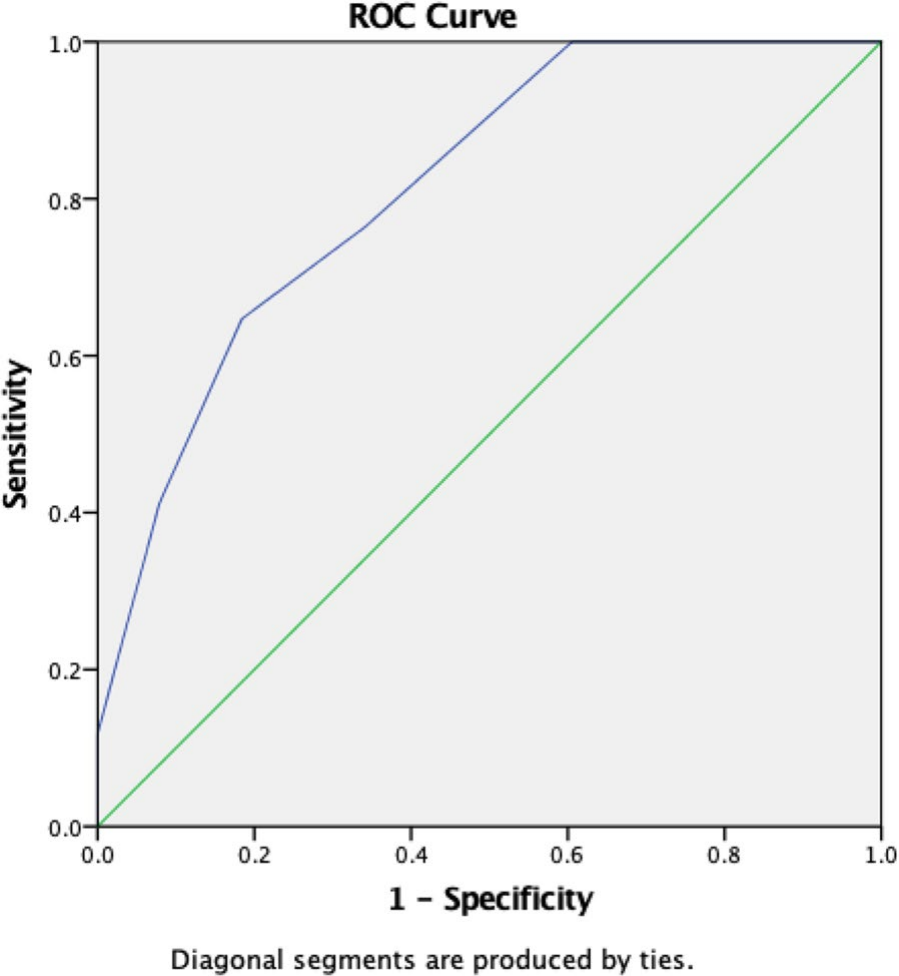
ROC curve for time from injury to reduction attempt to predict the success of closed reduction. *ROC* receiver operating characteristic

Selected parameters that might be related to the success rate of CR at the ED are shown in Table 2. There was a trend for older age (36.18 vs. 30.82, *p* = 0.19) and lower BMI (26.73 vs. 28.35, *p* = 0.48) in patients whose hips were reduced successfully compared with those who had failed CR at the bedside. However, neither one was statistically significant. There was no difference in sex, ISS, NISS, the presence of femoral head fracture, marginal impaction, and the size of the posterior wall fragment between the two groups. When comparing the TIR, the time interval was significantly shorter (2.24 vs. 4.11 h, *p* = 0.01) in patients whose hips were successfully reduced at the ED. Receiver operating characteristic (ROC) curve analysis was utilized to determine the cut-off level. Using time from TIR as a predictor of CR failure revealed an area under the curve (AUC) of 0.815 (Fig. 2). The optimal cut-off value was 3.5 h from injury using the Youden index.

**Table 2:** Group analysis for hip posterior fracture-dislocation receiving closed reduction at ED

	Reduction successful at bedside	Reduction failed at bedside	*p* value
Number	38	17	
Age (mean + SD) years	36.18 (SD 15.44)	30.82 (SD 12.97)	0.19
Sex (%)			
Male	32 (84.2%)	14 (82.4%)	0.86
Female	6 (15.8%)	3 (17.6%)	
ISS (mean + SD)	12.03 (SD 6.00)	11.65 (SD 6.46)	0.84
NISS (mean + SD)	18.92 (SD 7.19)	19.76 (SD 6.57)	0.67
BMI (mean + SD)	26.73 (SD 4.99)	28.35 (SD 6.98)	0.48
Marginal impaction (%)	13(34.2%)	5(29.4%)	0.97
Associated femoral head fracture (%)	16 (42.1%)	7 (41.2%)	0.95
Posterior wall fragment size (%, mean + SD)	35.55 (SD 18.66)	28.70 (SD 21.27)	0.27
Time from injury to first close reduction attempt (h, mean + SD)	2.24 (SD 1.30)	4.11 (SD 1.80)	0.01
Time to reduction (h, mean + SD)	2.24 (SD 1.30)	7.31 (DS 3.94)	0.01

Posterior wall fragment size was based on Moed’s method

*ISS* injury severity score, *NISS* new injury severity score, *BMI* body mass index, *ED* emergency department, *SD* standard deviation

For patients who received reduction attempts within 3.5 h from injury, the success rate of bedside CR at the ED was 86.2% (25/29). The success rate dropped to 50% (13/26) when the reduction attempt was beyond 3.5 h from injury, which is statistically significant (*p* < 0.01) (Table 3).

**Table 3:** Time from injury to first closed reduction attempt

	Within 3.5 h	Beyond 3.5 h	*p* value
Number	29	26	
Closed reduction			
Successful	25 (86.2%)	13 (50%)	*p* < 0.01
Failure	4 (13.8%)	13 (50%)	

## Discussion

Hip fracture-dislocation often results from high-energy trauma. Prompt reduction of a dislocated hip joint may decrease the incidence of late sequelae such as early-onset osteonecrosis of the femoral head and post-traumatic osteoarthritis [1–8]. The current consensus regarding the maximum time from injury for a successful reduction is within 6 h [9–11]. Several factors may interfere with the on-time successful reduction of a hip joint, such as additional time transferring from hospital to hospital, a complex fracture of the hip joint, concomitant injuries to organs in the torso, and the experience of the orthopedic surgeon who performs the procedure. Based on the literature, several CR maneuvers have been proposed for hip dislocation [15, 19–24]. We postulated that no single reduction maneuver was superior to others or suitable for all circumstances. A surgeon tends to choose a maneuver based on familiarity with the reduction maneuver and the patient’s actual presentation.

The only factor that was found to be potentially related to the success rate of bedside CR at ED was the TIR. The correlation between a delay in attempting reduction and a higher reduction failure rate has been documented in traumatic shoulder dislocation [25, 26]. To the best of our knowledge, this is the first article that has applied this association to traumatic hip dislocation. Using ROC curve analysis, we obtained an AUC of 0.815, and the cut-off value for successful reduction was 3.5 h from injury. Though we did not have strong proof regarding this point, we assumed that this finding might be related to the consequence of soft tissue reaction after hip trauma. When a hip joint is dislocated, periarticular muscles contract as a response to the traumatic force. Progressive tissue enema and swelling from cellular damage and hematoma formation may further increase the difficulty of CR. This may explain why CR is more difficult when the time interval from injury to reduction attempt is longer. Despite 6 h being a golden rule for successfully reducing a dislocated hip joint to prevent osteonecrosis, we found 3.5 h to be the success determinant for CR.

Since the TIR might play a crucial role in successful reduction, the place where the maneuver will be performed was a derived issue. The advantages of bedside reduction at the ED were its time-saving nature, cost-effectiveness, and the requirement of fewer medical resources [12–14]. However, inadequate sedation and muscle relaxation and the difficulty of performing fluoroscopy at the bedside might result in a failed reduction. On the contrary, reduction in the OT under general anesthesia prevented the above-mentioned problems, and OR could be promptly performed if CR failed. However, the waiting time required for the preparation of general anesthesia and OT settings may exceed the 6-h rule [27]. Though obesity and male sex have been proposed to be negative factors for CR [28], neither of these factors were observed in our study. Similarly, the presence of a femoral head fracture and small posterior acetabular wall fragment were not found in the analysis to be adverse factors for successful CR in our study. The only potential predictor for CR for a dislocated hip was TIR within 3.5 h. Based on our findings, if the TIR exceeds 3.5 h, we recommend that the OT should be prepared. Bedside CR at the ER can still be attempted, but the probability of success is much lower. If the initial CR fails, repeat hip manipulations should be avoided and the patient should be sent to OT for CR under general anesthesia.

The limitations of this study included its retrospective nature and small sample size. However, at a significance level of 0.05 and with a total sample size of 55, we had 84.2% power to detect the difference in the rate of failed CR at the ED. Additionally, only patients with hip posterior fracture-dislocation were enrolled. Other patterns of hip dislocations, such as a dislocated hip without fracture and anterior/obturator dislocation of the hip, were not assessed. Further studies should be conducted to explore if the 3.5-h rule fits all situations regarding dislocations.

## Conclusion

For patients with hip posterior fracture-dislocation, age, BMI, and associated femoral head fracture do not influence the success rate of bedside CR at the ED. TIR was a potential crucial factor for successful bedside CR. If the TIR exceeds 3.5 h, the probability of successful bedside CR at the ER deceases, and the OT should be prepared in case of failed bedside CR.

## Data Availability

The datasets used and/or analyzed during the current study are available from the corresponding author on reasonable request.

## Abbreviations

AP: Anteroposterior
AUC: Area under curve
BMI: Body mass index
CR: Closed reduction
ED: Emergency department
ISS: Injury severity score
NISS: New ISS
OR: Open reduction
OT: Operation theater
ROC: Receiver operating characteristic
TIR: Time from injury to first reduction attempt
